# Optimizing the Region for Evaluation of Global Gamma Analysis for Nasopharyngeal Cancer (NPC) Pretreatment IMRT QA by COMPASS: A Retrospective Study

**DOI:** 10.3389/fonc.2022.859415

**Published:** 2022-06-14

**Authors:** Wenli Lu, Ying Li, Wei Huang, Haixia Cui, Hanyin Zhang, Xin Yi

**Affiliations:** Department of Oncology, The First Affiliated Hospital of Chongqing Medical University, Chongqing, China

**Keywords:** patient-specific quality assurance, region for evaluation, global gamma passing rate, percentage dose error, action limits

## Abstract

**Background:**

The global gamma passing rate is the most commonly used metric for patient-specific pretreatment quality assurance in radiation therapy. However, the optimal region for evaluation and specific action limits (ALs) need to be explored. Therefore, this study was carried out to explore the optimal region for evaluation of the global gamma passing rate and define ALs by using the COMPASS software.

**Methods:**

A total of 93 intensity-modulated radiation therapy (IMRT) plans for nasopharyngeal cancer (NPC) patients, including 61 original plans and 32 multileaf collimator (MLC) error-introduced test plans, were selected for retrospective analysis. Firstly, the dose distribution was divided into six isodose regions (“≥10%”, “≥20%”, “≥30%”, “≥40%”, “≥50%”, and “≥60%”) based on the prescribed dose and one clinically oriented region for evaluation (“whole”) to perform the three-dimensional (3D) global gamma reanalysis. Meanwhile, the percentage gamma passing rate (%GP), mean gamma index (μGI) based on 3%/2 mm criteria, and percentage dose error (%DE) of the dose–volume histogram (DVH) metrics were recorded by COMPASS application. Secondly, the Pearson’s correlation coefficient was used to analyze the correlation between %GP and %DE and between μGI and %DE in different regions. Additionally, receiver operating characteristic (ROC) methodology was applied to quantify the fraction of patient-specific plans evaluated as “fail” and “pass”. In order to improve the correlation between gamma analysis result and clinical criteria, ROC analysis was carried out in accordance with hybridization analysis criteria (%DE ≤3%, %GP ≥90% and %DE ≤3%, μGI ≤0.6). ROC was performed for two purposes: 1) to analyze the sensitivity and specificity of %GP and μGI in different regions for evaluation and 2) to define the ALs of %GP and μGI in the optimal region for evaluation. Finally, the plans introduced with MLC errors were prepared for validation. Moreover, we also compared the positive rate of ALs of both %GP and μGI in detecting MLC error-introduced plans in different regions.

**Results:**

1) In our study, a number of DVH-based metrics were found to be correlated with the evaluation parameters. The corresponding number was 4, 2, 1, 1, 1, 1, and 3 in γ_whole_, γ_10%_, γ_20%_, γ_30%_, γ_40%_, γ_50%_, and γ_60%_, respectively, and 5, 3, 0, 1, 1, 4, and 2 in μGI_whole_, μGI_10%_, μGI_20%_, μGI_30%_, μGI_40%_, μGI_50%_, and μGI_60%_, respectively. The results by COMPASS have revealed that the %DE of specific structures has a slightly higher correlation with both %GP and μGI of the “whole” region compared with that of any other region. However, it is a moderate correlation (0.5 ≤ |r| < 0.8). 2) The areas under the curves (AUCs) of γ_whole_, μGI_whole_, μGI_40%_, μGI_50%_, and μGI_60%_ were >0.7 based on 3%/2 mm criteria. According to the Youden coefficient, we defined the ALs of γ_whole_ ≥92%, μGI_whole ≤_0.36, μGI_40%_ ≤0.43, and μGI_60%_ ≤0.40 based on 3%/2 mm criteria. 3) In the validation, for original plans, the accuracy of AL_γwhole_, AL_γ10%_, AL_μGIwhole_, AL_μGI40%_, AL_μGI50%_, and AL_μGI60%_ was 23%, 9.8%, 90%, 80.3%, 9.8%, and 88.5%, respectively. For test plans with systematic MLC errors smaller than 0.8 mm, the positive rates of AL_γwhole_, AL_γ10%_, AL_μGIwhole_, AL_μGI40%_, AL_μGI50%_, and AL_μGI60%_ were 25%, 58%, 92%, 92%, 42%, and 100%, respectively. For the plans with systematic MLC errors higher than 0.8 mm, the positive rates of all AL_%GP_ and AL_μGI_ were 100%. From the COMPASS validation results, the accuracy of γ_whole_, μGI_whole_, μGI_40%_, and μGI_60%_ was higher than that of the conventional γ_10%_ and commonly used μGI_50%_.

**Conclusions:**

Compared with the traditional evaluation region (i.e., the criteria with a threshold of 10% or a threshold of 50%, it was the same with the isodose regions of “≥10%”, “≥50%” based on the prescribed dose in our study), the “whole” region is more meaningful to the clinic by COMPASS. The accuracy of μGI_whole_ is higher than that of the conventional γ_10%_ and the commonly used μGI_50%_.

## Introduction

As an advanced radiation therapy technique, intensity-modulated radiation therapy (IMRT) allows for a complex dose distribution, with sharp dose gradients often achieved at the edge of the planning target volume (PTV). As IMRT is able to create an intentionally inhomogeneous dose distribution, it has routinely been employed as a radiotherapy technique (1). Due to the complex features of the inverse planning and the complexity in the delivery process, safety and quality assurance (QA) are important (2). As an essential and important process, patient-specific QA analyzes and evaluates the deviation between the predicted dose and the delivered/measured dose. This process can be carried out by using film dosimetry, ionization chambers, electronic portal imaging device (EPID), two-dimensional (2D) array detector, three-dimensional (3D) dosimetric system, gel dosimetry, log file, etc. (3, 4). Introduced in a seminal work by Low et al. (5), the global gamma index method is the most widely used technique for comparison of dose distribution for patient-specific pretreatment QA in radiation therapy (5–7). The 2D or 3D gamma analysis could be chosen based on different equipment. As a 3D volume analysis, the 3D gamma analysis can be used by COMPASS, Delta^4^, ArcCheck, Dolphin, OCTAVIUS 4D, etc. As a plane analysis, the 2D gamma analysis is often used by array detector, just like EPID, log file, Mapcheck, etc. Compared with the 2D gamma analysis, the 3D gamma analysis is increasingly winning high favor among medical physicists because it can provide more comprehensive QA information. However, the 3D gamma analysis could make the Region of Interest (ROI) selection more complex.

In clinical practice, the results of the patient-specific QA should reveal the percentage dose errors (%DEs) of dose–volume histogram (DVH) metrics (i.e., if the results of the patient-specific QA was acceptable, the %DE should be ≤3%), which are the important parameters used by radiation oncologists (8). However, Nelms et al. (9), Stasi et al. (10), and Fredh et al. (11) have found that the conventional global gamma analysis has limited sensitivity to dose errors of DVH metrics. Recommended by the TG-218 (3), the conventional global gamma analysis for QA measurements and corresponding treatment plan was performed in absolute dose mode, adopting global normalization (often using the maximum planned dose point) and a dose threshold of 10%. In some studies, it has been argued recently that the percentage gamma passing rate (%GP) of individual structures is more sensitive to dose errors than that in the conventional gamma method (12, 13). The %GP of individual structures provides a greater insight into the dose delivered to target volume and organs at risk (OARs). Stasi et al. (10), Heilemann et al. (12), and Yi et al. (13) have adopted the %GP of individual structures instead of conventional global gamma. Because the %GP of individual structures can not only improve the weak correlation between the %GP and %DE but also provide spatial information of dose errors that are unavailable in conventional gamma analysis. However, this method is only suitable for plans for one body site with a simple anatomy just like the IMRT plan for cervical cancer. It is difficult to be promoted in head and neck plan verification due to multiple targets and OARs that cause inefficiencies and increased clinical workload due to an abundance of DVH parameters that must now be reviewed.

In some reports (14), the current IMRT QA practice has been fundamentally questioned due to the poor sensitivity and specificity of the global gamma index implementation. Under such circumstances, Stojadinovic et al. (15) and Yu et al. (6, 16) have proposed the divide and conquer (D&C) gamma method. In this method, the dose distribution is divided into distinct regions: i) a high-dose (HD) region within the 90% isodose, ii) a high-gradient (HG) region with doses ranging from 50% to 90% isodose, iii) a medium-dose (MD) region with doses ranging from 20% to 50% isodose, and iv) a low-dose (LD) region with doses ranging from 10% to 20% isodose. It has been indicated that the conventional gamma method includes almost all dose regions (≥10% isodose level). However, from a clinical perspective, not all dose regions are equally essential. By using the D&C gamma method, more information can be provided for validation without further increasing the time-intensive nature of IMRT QA. Hence, it has challenged the adequacy of the conventional approach to patient-specific QA analysis. However, these publications have neither illustrated the correlation between the QA results and clinical DVH metrics nor verified the sensitivity and specificity of the D&C gamma method.

In addition, the %GP has been explored in most studies, but the mean gamma index (μGI) has been accounted for in only a few publications. In fact, Stock et al. (17) have proposed the initial concept of achieving the μGI action limits (ALs) for the hot area (receiving 50% or more of the prescribed dose) as early as in 2005. Recently, Visser et al. (18) have completed the 3D dose verification by combining μGI evaluation with %DE. Few publications have explored the relationship between μGI and %DE or the impact of different evaluation regions on μGI.

In this study, a measurement-guided dose calculation system was used to divide the measured dose distribution into six isodose regions and one global clinically concerned region (PTV plus all OARs). By doing so, we aimed to investigate the optimal region where the correlation between %GP and %DE and between μGI and %DE can be improved. Besides, this study was also implemented to prove a new method that does not further increase the time-intensive nature of IMRT QA.

## Materials and Methods

We selected 93 IMRT plans for nasopharyngeal cancer (NPC), including 61 original plans and 32 multileaf collimator (MLC) error-introduced test plans for retrospective analysis. These plans had been performed with measurement-based patient-specific QA. With ALs based on 3%/2 mm (global normalization) criteria recommended in American Association of Physicists in Medicine (AAPM) TG-218 (3), these IMRT QA plans were reanalyzed by using the different regions for evaluation.

### Definition of Regions for Evaluation

**Figure 1:**
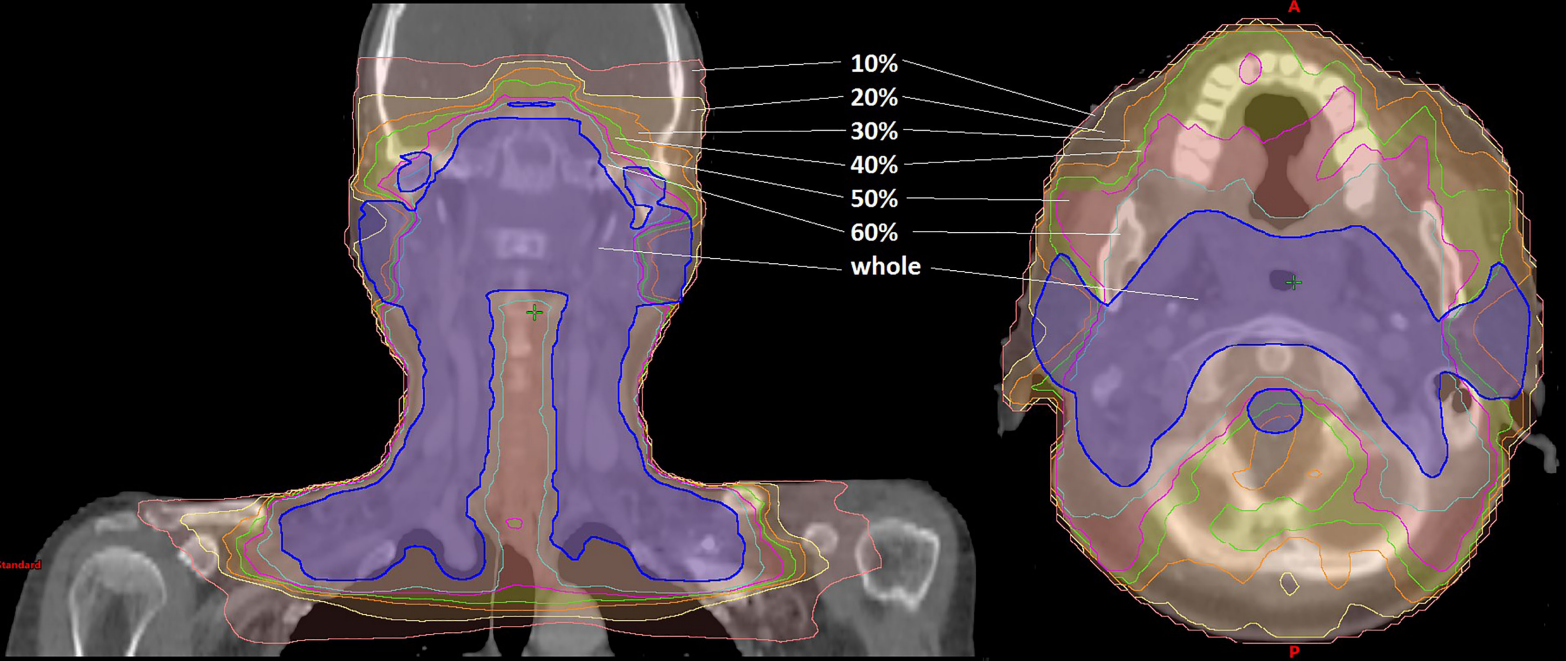
Dose distribution of the IMRT plan for nasopharyngeal cancer. The dose distribution was divided into seven parts depicted in different colors: the pink line refers to the region of “10%” label; the yellow line refers to the region of “20%” label; the orange line refers to the region of “30%” label; the green line refers to the region of “40%” label; the magenta line refers to the region of “50%” label; the cyan line refers to the region of “60%” label; the blue line refers to the region of “whole” label.

For the treatment planning, the dose distribution was computed with the anisotropic analytical algorithm (AAA). The dose calculation was carried out on a 2.5 mm isotropic dose grid in Eclipse treatment planning system (TPS) (Eclipse 13.5, VARIAN, USA) for a 6-MV photon beam on a UNIQUE linac (VARIAN, USA) equipped with a 120-leaf multileaf collimator (MLC) (millennium120 MLC, VARIAN, USA). In the dose verification, the structures were categorized into target volume and OAR as shown in **Table 1**. **Figure 1** shows the dose distribution that was divided into six isodose regions (“10%”, “20%”, “30%”, “40%”, “50%”, and “60%”) and clinically oriented region (“whole”):

(1) The region of “10%” label refers to all the voxels with a dose value of 10% or greater. Universal ALs adopted in AAPM TG-218 report: gamma passing rate ≥90% based on 3%/2 mm and a 10% dose threshold. Therefore, the region of “10%” label corresponds to the most widely used region by the conventional global gamma method.
(2) The region of “50%” label refers to all the voxels with a dose value of 50% or greater. It is the same region with the commonly used μGI for the hot area (receiving 50% or more of the prescribed dose) in the patient CT based on Stock et al. (17).
(3) The regions of “20%”, “30%”, “40%”, and “60%” labels refer to all the voxels with a dose value of 20%, 30%, 40%, and 60% or greater, respectively.
(4) The region of “whole” label indicates all voxels of PTV plus all of the OARs (spinal cord, brain stem, parotid L/R).

**Table 1:** The correlation between the %DE and the %GP in different regions for NPC patients.

		PTV			GTV			Parotid L	Parotid R	Brainstem	Spinalcord
		D_95_	D_mean_	D_5_	D_98_	D_mean_	D_2_	D_mean_	D_mean_	D_1_	D_1_
γ_whole_	r	-0.063	-0.241	-0.251	-0.014	-0.516	-0.606	-0.412	-0.457	0.236	0.148
	*p*	0.632	0.062	0.051	0.917	<0.001*	<0.001*	0.001*	<0.001*	0.067	0.254
γ_10%_	r	-0.064	0.055	0.067	-0.057	0.232	0.256	-0.085	0.185	-0.436	0.058
	*p*	0.625	0.674	0.609	0.665	0.072	0.046*	0.516	0.153	<0.001*	0.657
γ_20%_	r	-0.086	0.091	0.048	-0.083	0.184	0.147	-0.165	0.081	-0.393	0.047
	*p*	0.509	0.487	0.714	0.523	0.157	0.257	0.205	0.536	0.002*	0.716
γ_30%_	r	-0.096	0.064	-0.003	-0.101	0.102	0.021	-0.231	-0.037	-0.32	0.045
	*p*	0.462	0.622	0.979	0.439	0.433	0.87	0.074	0.777	0.012*	0.732
γ_40%_	r	-0.128	0.034	-0.053	-0.124	0.015	-0.112	-0.271	-0.148	-0.232	0.032
	*p*	0.325	0.797	0.685	0.341	0.911	0.391	0.035*	0.256	0.072	0.809
γ_50%_	r	-0.12	-0.041	-0.114	-0.079	-0.067	-0.196	-0.286	-0.223	-0.155	0.08
	*p*	0.357	0.755	0.383	0.545	0.61	0.129	0.025*	0.084	0.233	0.538
γ_60%_	r	-0.127	-0.083	-0.175	-0.076	-0.168	-0.281	-0.297	-0.255	-0.087	0.152
	*p*	0.331	0.527	0.177	0.562	0.196	0.028*	0.020*	0.048*	0.504	0.242

“*” indicates a significant difference, p < 0.05. D98, D95, D5, and D1 refers to the dose received by 98%, 95%, 5% and 1% volume of the structures including PTV, GTV, Parotid gland, Brainstem, Spinalcord, respectively. Dmean refers to the mean dose of the structures.

### Introduction of Multileaf Collimator Errors in Test Plans

**Figure 2:**
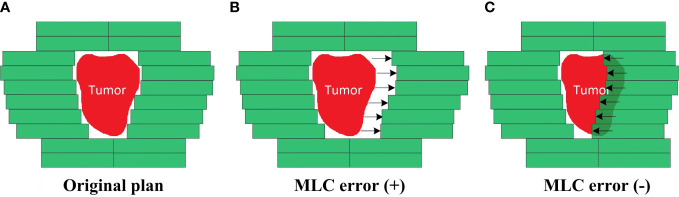
Creation of the MLC error-introduced IMRT plan. **(A)** Original plan. **(B)** Plan introduced with positive MLC errors. **(C)** Plan introduced with negative MLC errors.

An in-house Python program based on Pydicom (version 2.1.2) was used to introduce systematic MLC errors into the test plans by manipulation of DICOM RT files. As described in **Figure 2**, the introduced systematic MLC errors included an increase and a decrease of the distance between leaf pairs in the beam field. The magnitudes of systematic MLC errors were ±0.2, ± 0.4, ± 0.6, ± 0.8, ± 1.0, ± 2.0, ± 3.0, and ±4.0 mm, respectively. If any magnitude of MLC errors had led to a negative leaf gap in some of the leaf pairs, the gap of this leaf pair would have been set to 0.5 mm. After systematic MLC errors were introduced into the original plans, the modified RT files were reimported into TPS for dose calculation. A total of 32 plans were generated as a validation dataset.

### Quality Assurance Procedure

**Figure 3:**
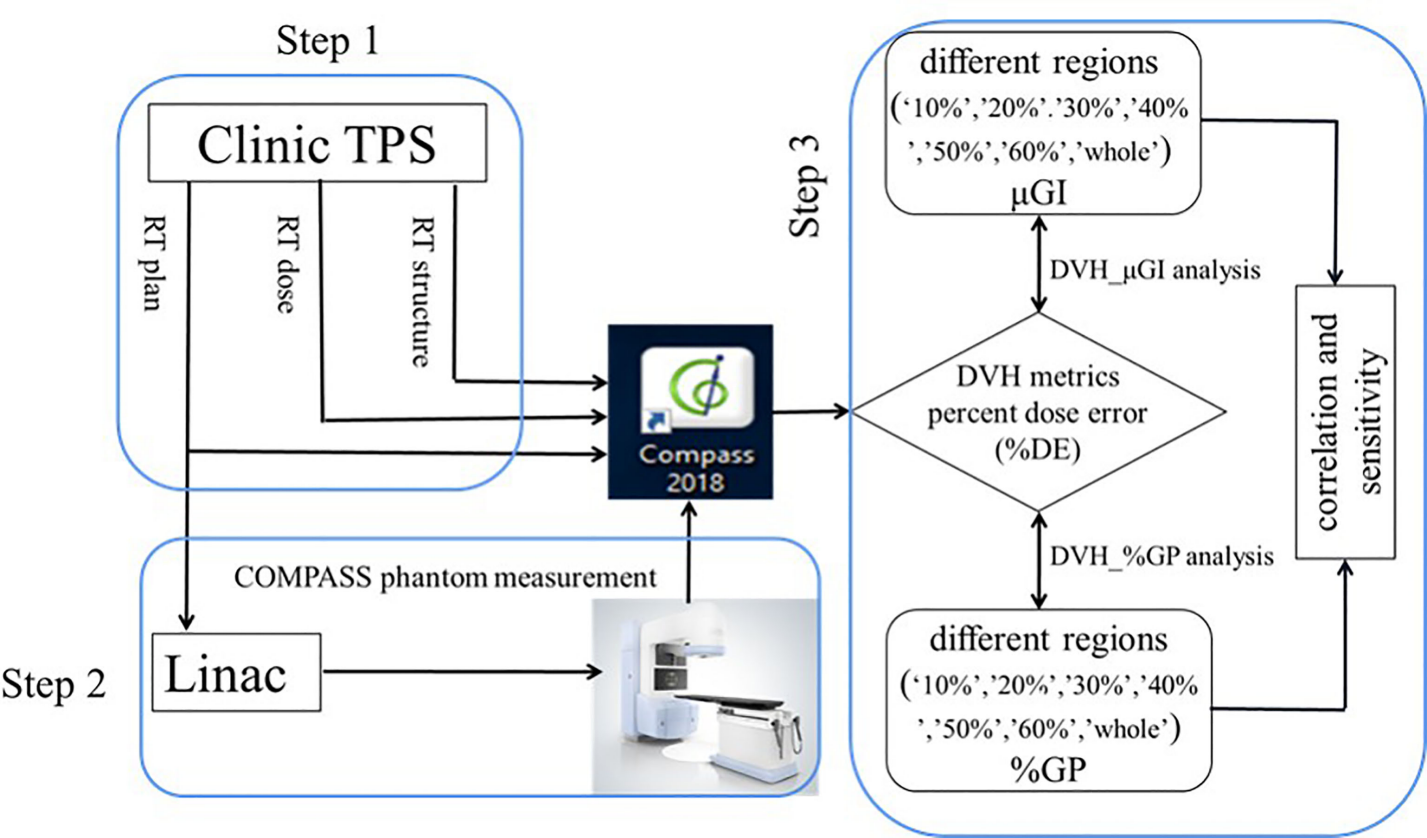
Flowchart of the quality assurance process in the study.

COMPASS (version 1.2. IBA Dosimetry, Germany) is a software that is used in conjunction with MatriXX^Evolution^ionization chamber-based 2D_IC array detector to provide a measurement-guided dose calculation. The COMPASS system is based on a beam model that includes the characteristics of the accelerator, a collapsed-cone convolution (CCC) algorithm. This allows pretreatment dose verification on CT images, including DVH. A strict commissioning of the COMPASS system, including the validation of accuracy for 2D_IC array (MatriXX, IBA Dosimetry) measurement, beam modeling, gantry angle sensor, and absolute dose calibration, was performed in advance according to the same standards as the clinic- used TPS. **Figure 3** shows the flowchart of QA procedure that had been carried out in the study: i) Initially, all plans were transferred from the TPS to the COMPASS software, including all of the DICOM files (RT plans, RT doses, RT structures, and CT images). ii) Afterward, all plans were performed by a linear accelerator to obtain the dose fluence by COMPASS so as to get the measurement-based QA. Meanwhile, the MatriXX was hung under the collimator to obtain the dose fluence. iii) Finally, the 2D_IC array measurement doses were reconstructed on patients’ CT images using the COMPASS application that models the characteristics of the linear accelerator head.

Furthermore, the dose distribution was calculated by the CCC algorithm and on a 2.5 mm isotropic dose grid by using the COMPASS application. In comparing the treatment planning and measurement-based QA, we recorded %DE, %GP, and μGI in different regions for reanalysis.

i) The %DE between the TPS dose and DVH index of measurement-based QA for specific structures was calculated as follows:


(1) $$\%DE_{i}=\left|\frac{D_{(i)eval}-D_{(i)ref}}{D_{(i)ref}}\right|\times 100\%$$

where D_eval_ is the measurement-based QA dose acquired by COMPASS, D_ref_ refers to the treatment planning dose, and *i* refers to the measured and calculated dose point pair.

ii) The global %GP is defined as follows:


(2) $$\Gamma_{i}\left(r_{ref},r_{eval}\right)=\sqrt{\frac{\Delta d^{2}}{\delta d^{2}}+\frac{\Delta D^{2}}{\delta D^{2}}}$$

(3) $$\gamma (r_{eval})=\text{min}\left\{\Gamma (r_{ref},r_{eval})\right\}\forall \left\{r_{eval}\right\}$$

The γ function is the minimum value of generalized Γ function computed for arbitrary isodose point distance Δd and dose difference ΔD. Values of γ between 0 and 1 will indicate that the comparison is acceptable according to the dose and distance criteria. If γ(r_eval_) ≤1, it will indicate that the comparison result of r_ref_ is acceptable. If γ(r_eval_) >1, it will mean a failure in the comparison result of r_ref_. The percentage of passing points in the gamma distribution is referred to as gamma pass rate (or %GP), δD/δd refers to the criteria of 3%/2 mm, and *i* refers to the measured and calculated dose point pair.


(4) $$\Delta D_{i}=\left|\frac{D_{(i)eval}-D_{(i)ref}}{D_{(i)norm}}\right|\times 100\%$$

where D_eval_ is the measurement-based QA dose acquired by COMPASS, D_ref_ is the treatment planning dose, and D_norm_ refers to the normalized max dose point. Normalization plays a critical role in the interpretation of dose comparison results. Specifically in global normalization, the dose difference between any measured and calculated dose point pair is normalized using the same value for all point pairs, often the maximum planned dose point. Here, *i* refers to the measured and calculated dose point pair.


(5) $$\Delta d_{i}=\left|r_{(i)eval}-r_{(i)ref}\right|$$

where *Δd* refers to the distance difference of the same dose between the measurement-based QA dose acquired by COMPASS and the treatment planning dose; *i* refers to the measured and calculated dose point pair.

iii) The definition of μGI is as follows:


(6) $$\mu GI_{x}=\frac{\sum_{1}^{n}\gamma (r_{eval})}{n}$$

μGI_x_ refers to the mean γ (r_eval_) for the measured and calculated dose point pair in *x* region, and *n* refers to the number of the measured and calculated dose point pairs in *x* region. A lower μGI is better.

### Hybrid Analysis: Comprehensive Gamma-Based and Dose–Volume Histogram-Based Analysis

In conventional gamma analysis (i.e., 3%/2-mm global normalization and 10% dose threshold, hereafter referred to as γ_10%_), dimensionless gamma passing rate is adopted to combine the dose difference (DD) with the distance to agreement (DTA). The universal ALs of %GP ≥90% in AAPM TG-218 report was proposed.

The μGI (the gamma index for receiving 50% or more of the prescribed dose, hereafter referred to as μGI_50%_) was the gamma passing rate for the composite dose. It was obtained and categorized by a global 3D gamma analysis (17, 18). The μGI_50%_ was categorized into “PASS” (acceptable for treatment), “EVAL” (to be evaluated by the medical physicist), or “FAIL” (rejected for treatment). Criteria for the μGI_50%_ are classified into PASS (μGI_50%_ ≤0.4, acceptable for treatment), EVAL (0.4 < μGI_50%_ < 0.6, to be evaluated by the medical physicist), and FAIL (μGI_50%_ ≥0.6, rejected for treatment).

**Figure 4:**
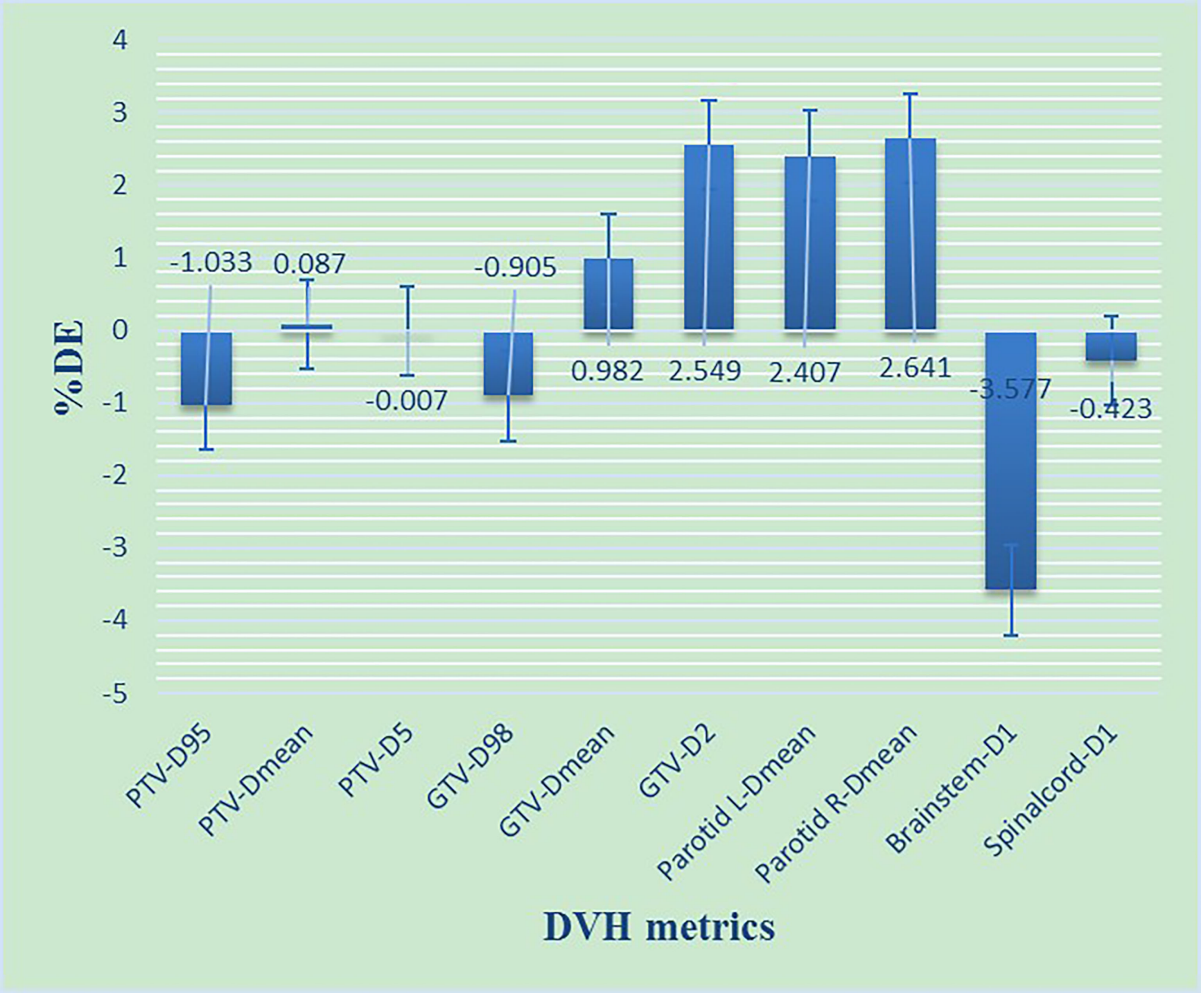
The %DE of DVH metrics based on 3%/2 mm criteria for NPC patients. D98, D95, D5, and D1 refers to the dose received by 98%, 95%, 5% and 1% volume of the structures including PTV, GTV, Parotid gland, Brainstem, Spinalcord, respectively. Dmean refers to the mean dose of the structures.

DVH information was acquired for the Planning Target Volume (PTV), gross tumor volume (GTV), and four types of OARs (brain stem, spinal cord, parotid L, parotid R). The details of the DVH metrics are shown in **Figure 4**. According to the study by Cozzolino et al. (19) and Visser et al. (18), the DVH ALs should be set to 2%–5%. Therefore, we have chosen DVH ALs of 3%. It means that the %DE of all the structures must be ≤3%.

Based on available literature (13, 18), combinations of DVH information, %GP, and μGI were employed in this study. By doing so, we aimed to improve the correlation between gamma analysis result and clinical criteria. In particular, a measured plan can be considered to be clinically acceptable when the following two conditions are met (16, 20): i) γ_10%_ ≥90% and the absolute value of %DE ≤3%; ii) μGI_50%_ <0.6 and the absolute value of %DE ≤3%.

### Data Analysis

**Figure 5:**
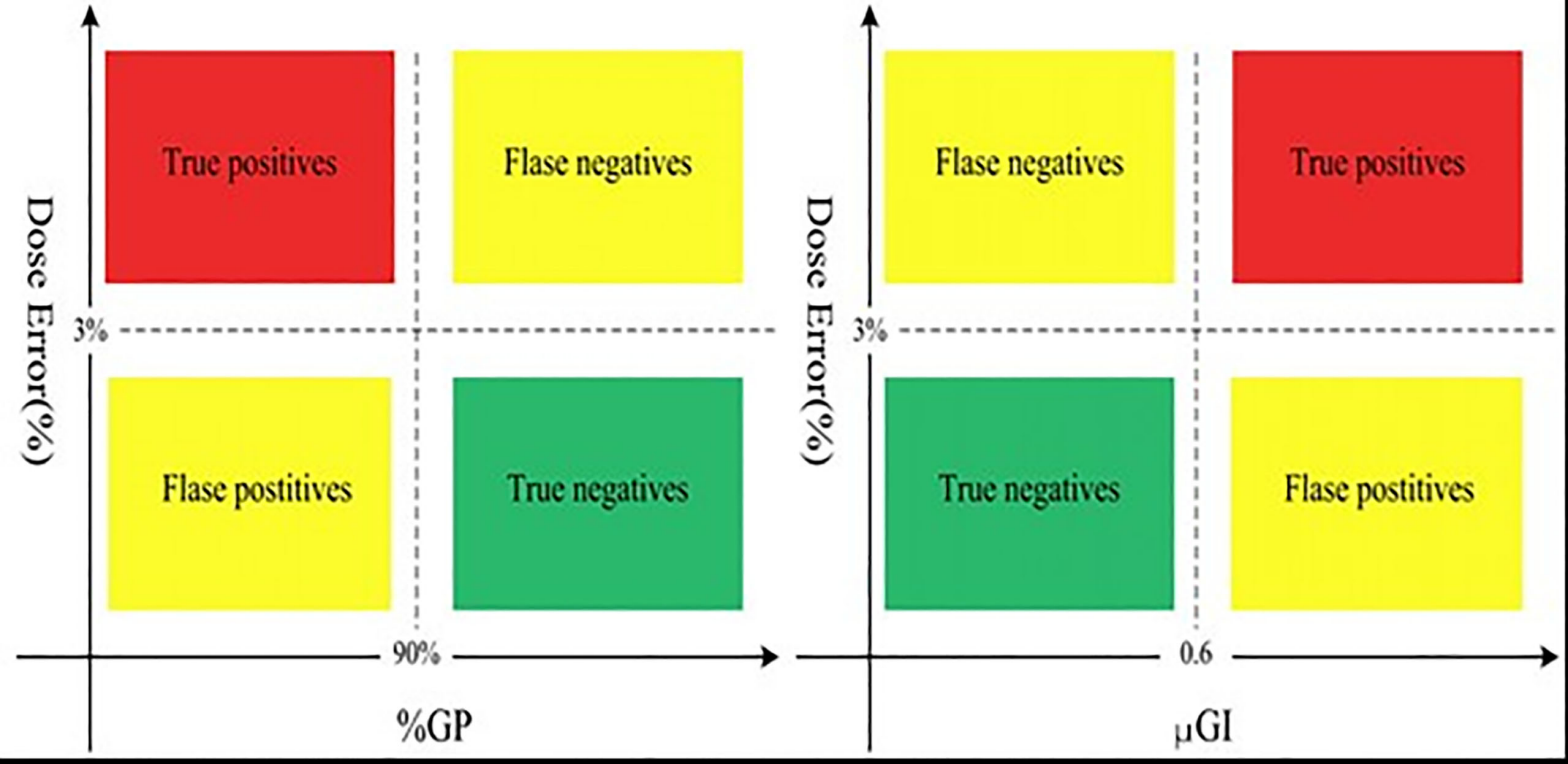
The DVH_%GP and DVH_μGI evaluation charts.

**Figure 5** shows the patient-specific QA reports of the 61 original IMRT plans that were categorized into four types based on the DVH_γ_10%_ or DVH_ μGI_50%_ ALs:

i) In DVH_γ_10%_ evaluation chart: “true negatives” (TNs) refer to γ_10%_ ≥90% and the absolute value of %DE ≤3%; “false negatives” (FNs) refer to γ_10%_ ≥90% and the absolute value of %DE >3%; “true positives” (TPs) refer to γ_10%_ <90% and the absolute value of %DE >3%; “false positives” (FPs) refer to γ_10%_ <90% and the absolute value of %DE ≤3%
ii) In DVH_ μGI_50%_ evaluation chart: “true negatives” (TNs) refer to μGI_50%_ <0.6 and the absolute value of %DE ≤3%; “false negatives” (FNs) refer to μGI_50%_ <0.6 and the absolute value of %DE >3%; “true positives” (TPs) refer to μGI_50%_ ≥0.6 and the absolute value of %DE >3%; “false positives” (FPs) refer to μGI_50%_ ≥0.6 and the absolute value of %DE ≤3%

When the correlation between %DE and %GP and between %DE and μGI in various regions was analyzed by the SPSS 19.0 (SPSS Inc., Chicago, IL, USA) software, we used Pearson’s correlation coefficient (r). If the *p* value was <0.05, it would have indicated that %DE has a correlation with both %GP and μGI. Different correlation coefficients (r) represent different degrees of correlation: If |r| ≥ 0.8, it will be considered to be highly correlated; if 0.5 ≤ |r| < 0.8, it will be considered to be moderately correlated; if |r| < 0.5, it will be considered to be weakly correlated.

In this study, the TP rate indicated the sensitivity and was defined as TP/(TP+FN), while the TN rate indicated the specificity and was given by TN/(TN+FP). The receiver operating characteristic (ROC) curves were generated based on the TN and TP rates to characterize %GP and μGI in different regions. The area under the curve (AUC) was used as the classification indicator as follows: If AUC = 1, it would have meant that the indicator can be used for classification precisely; If AUC = [0.8, 0.9], it would have indicated a very good effect; If AUC = [0.7, 0.8], it would have indicated an average effect; If AUC = [0.5, 0.7], it would have meant a low effect. Consequently, the ALs can also be defined according to the Youden coefficient (21). Youden coefficient is used to evaluate the authenticity of the screening test. Its value is equal to the sum of sensitivity and specificity of point in ROC minus 1. The higher the Youden coefficient, the better the authenticity. The value of the max Youden coefficient point in ROC is the cutoff.

Finally, in the validation, for the original plans and the MLC error-introduced test plans, the ALs were used to evaluate the accuracy of various regions.

## Results

### Dose–Volume Histogram Metrics Evaluation and 3D γ_Analysis

**Figure 6:**
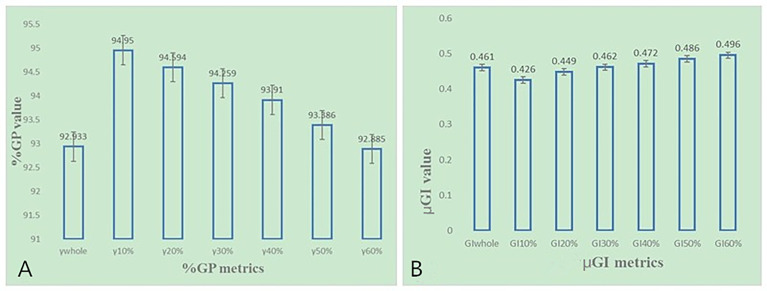
**(A)** The %GP in different regions based on 3%/2 mm criteria for NPC patients. **(B)** The μGI in different regions based on 3%/2 mm criteria for NPC patients.

As shown in **Figure 4**, most of the %DE metrics were found to be within 3% criteria, except for D_1_ of the brain stem. As shown in **Figure 6**, the larger the volume of the region, the smaller the results of %GP. However, the results of μGI were consistent with volume changing of the different regions. The %GPs of the different regions (γ_10%_, γ_20%_, γ_30%_, γ_40%_, γ_50%_, γ_60%_, and γ_whole_) were higher than 90%, and the μGIs of these regions (μGI_10%_, μGI_20%_, μGI_30%_, μGI_40%_, μGI_50%_, μGI_60%_, μGI_whole_) were lower than 0.6.

### Correlation Analysis Between %DE and %GP and Between %DE and μGI in Different Regions

Ten DVH-based parameters were chosen for evaluation. Correlation analysis between the different %DEs of DVH metrics and %GP, μGI of different regions was performed one by one as shown in **Table 1**. It shows that a number of DVH-based metrics were found to be correlated with the evaluation parameters (i.e., the corresponding number was 4, 2, 1, 1, 1, 1, and 3 in γ_whole_, γ_10%_, γ_20%_, γ_30%_, γ_40%_, γ_50%_, and γ_60%_, respectively). i) Only γ_whole_ is considered to be moderately correlated with the %DE of GTV-D_mean_ and GTV-D_2_ (0.5 ≤ |r| < 0.8, *p* < 0.05); ii) The γ_whole_, γ_10%_, γ_20%_, γ_30%_, γ_40%_, γ_50%_, and γ_60%_ are considered to be weakly correlated with the other %DE (|r| < 0.5, *p* < 0.05).

**Table 2** shows that a number of DVH-based metrics were found to be correlated with the evaluation parameters (i.e., the corresponding number was 5, 3, 0, 1, 1, 4, and 2 in μGI_whole_, μGI_10%_, μGI_20%_, μGI_30%_, μGI_40%_, μGI_50%_, and μGI_60%_, respectively). i) The μGI_whole_ is considered to be moderately correlated with the %DE of GTV-D_2_ (0.5 ≤ |r| < 0.8, *p* < 0.05); ii) The μGI_whole_, μGI_10%_, μGI_20%_, μGI_30%_, μGI_40%_, μGI_50%_, and μGI_60%_ are considered to be weakly correlated with the other %DE (|r| < 0.5, *p* < 0.05).

**Table 2:** The correlation between the %DE and the μGI in different regions for NPC patients.

		PTV			GTV			Parotid L	Parotid R	Brainstem	Spinalcord
		D_95_	D_mean_	D_5_	D_98_	D_mean_	D_2_	D_mean_	D_mean_	D_1_	D_1_
μGI_whole_	r	0.187	0.303	0.2	0.063	0.44	0.551	0.39	0.493	-0.149	-0.066
	*p*	0.149	0.018*	0.122	0.628	<0.001*	<0.001*	0.002*	<0.001*	0.253	0.613
μGI_10%_	r	0.136	-0.085	-0.068	-0.008	-0.299	-0.351	0.054	-0.151	0.524	0.063
	*p*	0.296	0.513	0.6	0.952	0.019*	0.005*	0.679	0.246	<0.001*	0.628
μGI_20%_	r	0.101	-0.005	0.028	0.189	-0.151	-0.176	0.051	0.052	0.247	0.01
	*p*	0.439	0.967	0.833	0.145	0.246	0.174	0.697	0.693	0.055	0.94
μGI_30%_	r	0.225	-0.053	-0.02	0.127	-0.218	-0.157	0.18	0.051	0.472	0.061
	*p*	0.082	0.685	0.88	0.33	0.092	0.226	0.164	0.699	<0.001*	0.641
μGI_40%_	r	0.245	0.019	0.002	0.149	-0.101	-0.012	0.248	0.169	0.343	0.076
	*p*	0.057	0.882	0.985	0.252	0.441	0.928	0.054	0.192	0.007*	0.561
μGI_50%_	r	0.256	0.078	0.042	0.114	-0.049	0.039	0.259	0.23	0.272	0.038
	*p*	0.046*	0.552	0.749	0.381	0.708	0.768	0.044*	0.075*	0.034*	0.77
μGI_60%_	r	0.276	0.134	0.103	0.076	0.066	0.124	0.257	0.247	0.232	-0.058
	*p*	0.031*	0.304	0.431	0.558	0.614	0.34	0.046*	0.055	0.072	0.658

“*” means a significant difference, p < 0.05. D98, D95, D5, and D1 refers to the dose received by 98%, 95%, 5% and 1% volume of the structures including PTV, GTV, Parotid gland, Brainstem, Spinalcord, respectively. Dmean refers to the mean dose of the structures.

### Receiver Operating Characteristic Analysis

**Figure 7:**
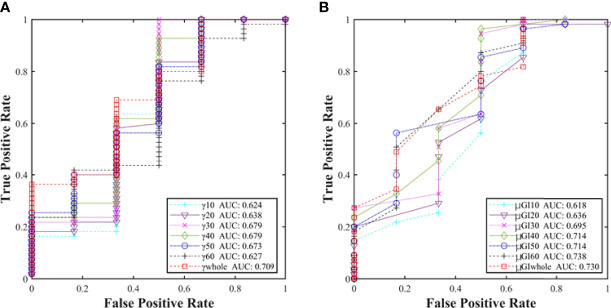
**(A)** Comparison of the AUCs of %GP in different regions (γ_whole_, γ_10%_, γ_20%_, γ_30%_, γ_40%_, γ_50%_, and γ_60%_) and **(B)** μGI (μGI_whole_, μGI_10%_, μGI_20%_, μGI_30%_, μGI_40%_, μGI_50%_, and μGI_60%_).

In accordance with the results, further analysis on sensitivity was performed by using the ROC of %GP (γ_whole_, γ_10%_, γ_20%_, γ_30%_, γ_40%_, γ_50%_, and γ_60%_) and μGI (μGI_whole_, μGI_10%_, μGI_20%_, μGI_30%_, μGI_40%_, μGI_50%_, and μGI_60%_). **Figure 7** shows the areas under the curves (AUCs) of %GP and μGI in different regions. The AUCs of both %GP and μGI acquired for the “whole” region were higher than 0.700. On the other hand, the AUCs of μGI_40%_, μGI_50%_, and μGI_60%_ were 0.714, 0.714, and 0.738, respectively. It would have meant that the %GP and the μGI of other areas have a very low efficiency in classification. Therefore, these parameters were chosen for further analysis.

### The Accuracy Analysis

According to the Youden coefficient, the ALs of the “whole”, “40%”, and “60%” regions were adopted for ALγ_whole_ ≥92%, AL_μGIwhole ≤_0.36, AL_μGI40%_ ≤0.43, and AL_μGI60%_ ≤0.40, respectively, based on 3%/2 mm criteria.

**Figure 8:**
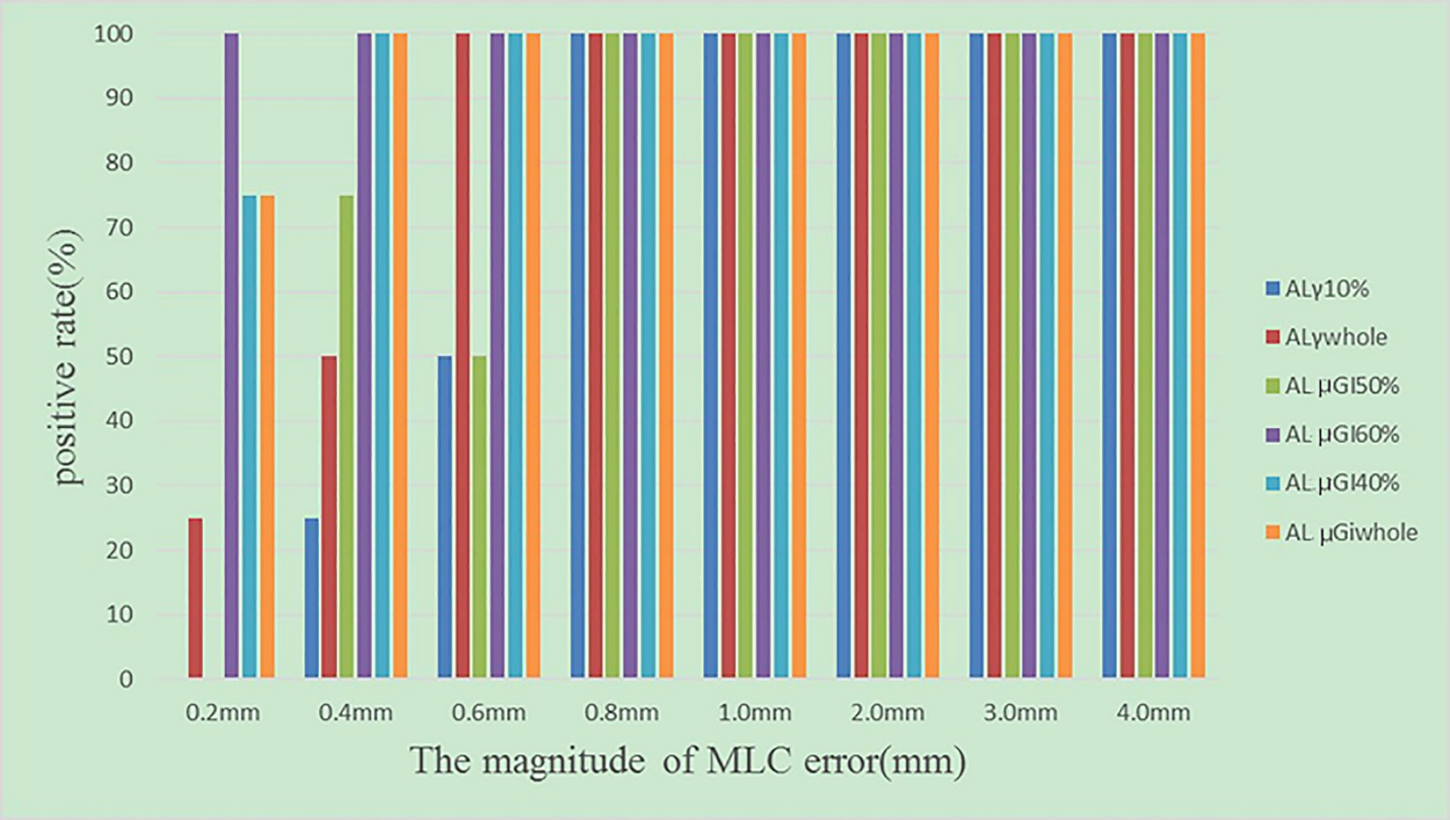
The positive rates of test plans introduced with different magnitudes of systematic MLC errors.

In the validation, for the original plans and the MLC error-introduced test plans, the accuracy of the various regions was evaluated by ALs and compared with the hybrid analysis. As shown in **Table 3**, for original plans, the accuracy of AL_γwhole_, AL_γ10%_, AL_μGIwhole_, AL_μGI40%_, AL_μGI50%_, and AL_μGI60%_ is 23%, 9.8%, 90%, 80.3%, 9.8%, and 88.5%, respectively. As shown in **Figure 8**, for test plans with systematic MLC errors smaller than 0.8 mm, the positive rates of AL_γwhole_, AL_γ10%_, AL_μGIwhole_, AL_μGI40%_, AL_μGI50%_, and AL_μGI60%_ were 25%, 58%, 92%, 92%, 42%, and 100%, respectively; for test plans with systematic MLC errors higher than 0.8 mm, the positive rates of all the AL_%GP&μGI_ in identifying MLC error-introduced plans were 100%.

**Table 3:** The comparison of the hybrid analysis among the evaluation parameters (i.e., AL_γ10%_, AL_γwhole_, AL_μGI50%_, AL_μGIwhole_, AL_μGI40%_, AL_μGI60%_).

		AL_γ10%_	AL_γwhole_	AL_μGI50%_	AL_μGIwhole_	AL_μGI40%_	AL_μGI60%_
Hybrid analysis	a	9.8%	23%	9.8%	90%	80.3%	88.5%
	b	91.2%	77%	91.2%	10%	19.7%	11.5%

a refers to the hybrid analysis results corresponding to the evaluation parameters; b refers to the hybrid analysis results that do not correspond to the evaluation parameters.

## Discussion

In global gamma analysis, all points are normalized to one reference dose point where the maximum dose or isocenter dose is the most common choice (9). In general, errors are masked in the lower dose regions in the conventional global gamma because the low dose points are normalized to a high dose point (22). Furthermore, the TG-218 report has also implied that a low dose region (such as 10% of the prescription dose) can significantly affect the analysis (3). As a result, the conventional global gamma passing rate is often questioned regarding its limitations or clinical relevance (23, 24). Therefore, changing the evaluation region for the global gamma analysis (or changing the minimum threshold) is a preferred method because it can decrease the bias of the global gamma analysis.

In essence, the methodology proposed in our study computed the %GP and the μGI of reduced sample voxels of the dose distribution in different regions rather than that of all voxels. A conventional gamma analysis includes all voxels in the dose distribution (with dose values of 10% or greater). However, not all voxels have clinical significance in patient-specific QA. The targets and OARs are critical clinically relevant volume for making a final determination. The “whole” region for evaluation was explored with increasing dose level until it only contained the targets and OARs. The correlation between %GP, μGI, and %DE of various dosimetry metrics was used to assess the relevance of both %GP and μGI with clinically relevant observations.

From calculation equations 3–6, it is μGI rather than %GP that was able to more or less reflect the gamma value change of each evaluated point. The ROC analysis also demonstrated that the μGI in different regions was more sensitive than %GP. Therefore, it has implied that μGI reveals more clinically relevant problems compared with %GP.

In this study, both %GP and μGI results of seven regions for evaluation were found to have moderate correlation with the DVH metrics. This can be compared with the studies made by Zhen et al. (25) and Chan et al. (26). The μGI_whole_ was recommended to replace the conventional γ_10%_ and μGI_50%_ in the study. There are two reasons why the “whole” region is superior. One, from equations 3–6, both the spatial information and %DE were included in %GP and μGI. However, the dose difference information regarding specific structures was unavailable, which made %GP and μGI difficult to explore for assessing clinical implications (26). As the proposed “whole” region in our study only focused on the area of clinical concern, it can partially solve this problem. Moreover, Yu et al. (11) have demonstrated that different dose levels might include some other information such as the accuracy of TPS calculation in low-dose regions (10%–20% dose region), the accuracy of MLC modeling (50% dose region), and the expected information regarding the validity of the source model including beam energy, focal spot size, and collimator feature (high-dose regions). The “whole” region not only was the clinically concerned region but also provided the information in different dose levels. Although the sensitivities of μGI_60%_ and μGI_whole_ were similar, the “whole” region included more dose levels. Hence, it provided greater insight into the dose delivered to specific structures. According to the study by Cozzolino et al. (19) and Visser et al. (18), the DVH ALs should be set to 2%–5%. Therefore, we chose the DVH ALs of 3%. However, in areas with a sharp dose gradient and in metrics with small volumes, relatively high dose differences such as point dose (D_1_) were observed. For results whose %DE exceeded DVH ALs, it was acceptable from the clinical point of view if the measured dose of the target volume was higher than the calculated dose or if the measured dose of OAR was lower than the calculated dose (27). The reason was that the results would not affect clinical efficiency. This view was proven by the %DE of the brain stem in **Figure 5**. The %DE of brain stem (D_1_) of 32 cases was >3%, but most of the dose deviations were negative. Hence, this DVH ALs were too strict for OARs near the target volume such as the brainstem within or near the PTV in the NPC plan. The DVH ALs had an influence on defining the ALs of both %GP and μGI directly. So, if values determined for μGI_whole_ exceed the ALs, we need to check whether they have a clinical impact.

MLC errors mainly include random MLC positional errors and systematic positional errors. Random MLC positional errors have little dosimetric effect on IMRT plans (28). Steers and Fraass (23) concluded that the shifts in different directions may be an indication of different types of systematic errors arising from TPS model issues, machine issues, or other device issues. In addition, in our study, for the systematic MLC errors smaller than 0.8 mm, the positive rate of μGI_whole_ was higher compared with 0.75 mm systematic MLC errors in some publications (29), which were claimed to be undetected due to the poor resolution of array detectors. The author analyzed and concluded that the reason was that it was too strict to set the DVH ALs of 3% for the NPC plan. However, there is no consensus on the tolerance of the systematic MLC errors for patient QA. Numerous publications (30–32) have found that the systematic MLC errors up to 1 mm can produce a clinically relevant influence on the dose distribution. However, Rangel et al. (33) have suggested that systematic MLC errors need to be limited to 0.3 mm. Oliver et al. (34) have suggested that the systematic MLC errors should be within 0.63 mm to keep the PTV95 within 2%. In brief, μGI_whole_ can improve the detecting rate of small systematic MLC errors and the clinical effect of radiotherapy.

By gamma analysis, the verification results can be compressed into a single value (%GP or μGI). It can improve the efficiency of dose verification, which is usually important for clinics under a heavy workload. As proposed in this study, μGI_whole_ would not increase the clinical workload. Instead, it has a better correlation with DVH metrics than other commonly used evaluation metrics. Of course, these results also highlighted major limitations of our study. It was not a multicenter study and can potentially bias the analysis. In addition, these results may also have been affected by the specific choice of dosimeter, QA equipment, delivery system, and tumor sites (23). However, this study could be extended by stratifying the “whole” region by isodose level and anatomical site to provide more clinical information without increasing clinical work.

## Conclusion

The empirical-type metrics, such as those in the patient-specific QA, may vary from institution to institution, since those metrics are based on local equipment, processes, and treatment types. Therefore, the conventional regions including “10%” and “50%” may not be suitable for all institutions. Different regions of the global gamma analysis have different diagnostic efficiencies. The results of μGI_whole_ can provide a new metric for the global gamma analysis.

## Data Availability Statement

The original contributions presented in the study are included in the article/supplementary material. Further inquiries can be directed to the corresponding author.

## Ethics Statement

This study was reviewed and approved by the Ethical and Scientific Committees of The First Affiliated Hospital of Chongqing Medical University (Chongqing, China). Written consent was obtained from the patient individual(s) for the publication of any potentially identifiable images or data included in this paper.

## Author Contributions

WL, YL, and XY conceived and designed the study and contributed to the analysis and interpretation of trials and literature and development and review of the article. HC and HZ collected the data, reviewed literature, and wrote the paper. WH prepared the figures and participated in the revision of literature. All authors contributed to the article and approved the submitted version.

## Funding

This work was sponsored by Natural Science Foundation of Chongqing, China (cstc2021jcyj-msxmX0138).

## Conflict of Interest

The authors declare that the research was conducted in the absence of any commercial or financial relationships that could be construed as a potential conflict of interest.

## Publisher’s Note

All claims expressed in this article are solely those of the authors and do not necessarily represent those of their affiliated organizations, or those of the publisher, the editors and the reviewers. Any product that may be evaluated in this article, or claim that may be made by its manufacturer, is not guaranteed or endorsed by the publisher.

## Abbreviations

NPC, Nasopharyngeal cancer; IMRT, intensity-modulated radiation therapy; MLC, multileaf collimator; %GP, gamma passing rate; μGI, mean gamma index; %DE, percentage dose error; DVH, dose–volume histogram; ROC, receiver operating characteristic; AUCs, areas under the curves; AL, action limit; PTV, planning target volume; QA, quality assurance; EPID, electronic portal imaging device; 2D, two-dimensional; 3D, three-dimensional; D&C, divide and conquer; HD, high-dose; HG, high-gradient; MD, medium-dose; LD, low-dose; OARs, organs at risk; AAA, anisotropic analytical algorithm; TPS, treatment planning system; CCC, collapsed-cone convolution; DD, dose difference; DTA, distance to agreement; TN, true negative; FN, false negative; TP, true positive; FP, false positive.
